# Accessing Altered Metabolic Profile in Acute Deep Vein Thrombosis Through Nuclear Magnetic Resonance Spectroscopy

**DOI:** 10.3390/ijms262311345

**Published:** 2025-11-24

**Authors:** Letícia Queiroz da Silva, Thyerre Santana da Costa, Lucas Gelain Martins, Silmara Aparecida de Lima Montalvão, Stephany Cares Huber, Sandra Martins Silva Soares, Ljubica Tasic, Joyce Maria Annichino-Bizzacchi

**Affiliations:** 1 Hemocentro, Haemostasis and Inflammation Laboratory, Universidade Estadual de Campinas—UNICAMP, Campinas 13083-970, SP, Brazil; queiroz.leticia1994@gmail.com; 2 Biological Chemistry Laboratory, Institute of Chemistry, Universidade Estadual de Campinas—UNICAMP, Campinas 13083-862, SP, Brazil; t224944@dac.unicamp.br (T.S.d.C.); lucasgm@unicamp.br (L.G.M.); 3 Hemocentro, Haemostasis Laboratory, Universidade Estadual de Campinas—UNICAMP, Campinas 13083-970, SP, Brazil; silmara@unicamp.br (S.A.d.L.M.); huber@unicamp.br (S.C.H.); sandragr@unicamp.br (S.M.S.S.)

**Keywords:** deep vein thrombosis, metabolites, metabolomics, profiling, venous thromboembolism

## Abstract

Deep venous thrombosis (DVT) is characterized by the formation of a thrombus within deep veins. The unmet need to identify new biomarkers and causal risk factors in DVT patients has led to the use of novel techniques, such as metabolite analyses. This study aimed to characterize metabolic alterations in acute DVT patients using ^1^H-NMR spectroscopy and determine the persistence of these changes over a six-month follow-up. Metabolomics, particularly ^1^H-NMR spectroscopy, was performed on serum samples from acute DVT patients (first 30 days from diagnosis) and healthy controls (HC). Additionally, 10 plasma markers were evaluated using a Luminex kit. A total of 30 patients, with a mean age of 44 ± 12.5 years, primarily women (9 males:21 females), were included. Acute DVT patients showed elevated inflammatory markers, such as IL-6, IL-8, PDGF-AB/BB, and P-selectin, which later decreased in the follow-up group. However, adhesion molecules like sVCAM-1 and sICAM-1 have increased after six months. Metabolomics analysis revealed significantly decreased levels of glutamine, glucose, and branched-chain amino acids (BCAAs), alongside increased lactate levels in acute DVT samples. Metabolomic profiles showed only partial normalization at follow-up, indicating persistent metabolic dysregulation. Overall, the reduced glucose metabolism and increased lactate levels indicate anaerobic metabolism, likely caused by tissue hypoxia due to impaired blood flow. Glutamine, essential for DNA, ATP, and protein synthesis, was notably reduced, potentially impairing endothelial cell proliferation and vascular repair. The presence of adhesion molecules in the follow-up group confirms persistent endothelial dysfunction. These findings suggest that metabolic and endothelial alterations may persist long after acute inflammation resolves in DVT patients. In conclusion, the persistence of metabolic dysregulation suggests chronic metabolic stress in these patients, potentially resulting from ongoing endothelial damage, low-grade inflammation, or altered mitochondrial function due to past tissue hypoxia.

## 1. Introduction

Deep venous thrombosis (DVT) is characterized by the formation of a thrombus within deep veins and is more frequent in the lower limbs [1]. In its acute phase, the main complication is the possible detachment of the thrombus or part of it, which can migrate to the pulmonary vessels and cause pulmonary embolism (PE)—an independent predictor of reduced survival [2]. DVT and PE comprise venous thromboembolism (VTE), one of the most important public health problems.

In numbers, VTE has an estimated annual incidence of 0.5 to 2 cases per 1000 individuals in the general population [3,4], being predominant in older age groups (4 to 8 cases over 60 years). Epidemiological studies indicate VTE as the third leading cause of death associated with cardiovascular diseases, after myocardial infarction and stroke [5]. However, this number might be higher, since low-income countries lack notification of VTE cases and their complications.

DVT is a multicausal disease in which the interaction of genetic and acquired risk factors can trigger the thrombotic process. However, approximately 30% of patients had no obvious risk factor identified, a fact that suggests the need to assess the presence of additional mechanisms still unknown in the etiology of the disease [6,7,8].

The identification of some biomarkers as effective diagnostic tools and possible therapeutic targets has been established in the past few years, for example, the evaluation of D-dimer as an ongoing fibrinolytic process with a negative predictive value [9,10]. However, the D-dimer assay has high sensitivity but low specificity, as this marker may be increased in situations such as infection, pregnancy, and post-surgery, among others [11,12].

The unmet need to find new biomarkers and causal risk factors in these patients has resulted in the use of novel techniques, such as metabolite analyses. This field seeks an analytical description of complex biological samples, aiming to quantify and characterize the end products of cellular metabolism, and allows us to understand the systemic changes in complex multicellular systems [13].

While studies with animals are well described, metabolomics with DVT human samples are not that common. A previous study demonstrated alterations in serum metabolic profile, glucose, lipids, unsaturated lipids, and glycoprotein A in DVT cohorts up to 2 years after thrombosis [14]. Whereas a large prospective case for the investigations of the risk of incident VTE, C5 carnitine was highlighted, and diacylglycerols were enriched in both VTE and pulmonary embolism-suffering individuals [15]. However, metabolites may be measured several years before a VTE event and may not represent the pathophysiologic state just preceding the VTE [16].

To date, only one study has investigated the role of potential metabolites in VTE during the acute phase. This study identified glutamine and glutamate in plasma samples, as well as adenosine 3′,5′-diphosphate, glutathione, and adenine in red blood cells, as metabolites potentially involved in VTE pathophysiology [17]. However, a key limitation of the study was the use of patients with clinical suspicion of VTE as the control group. The presence of symptoms in these individuals likely reflects underlying metabolic and inflammatory alterations, making comparisons with healthy individuals more robust.

In addition, it is important to understand if the metabolites’ pattern is found in the acute phase, and it is maintained even after 6 months of the diagnosis. Clinically, it is debated that the use of the risk–benefit of anticoagulation, bleeding, and DVT recurrence and follow-up research could help to improve their treatments.

In this context, this study aims to compare patients with DVT in the acute state with healthy individuals using a metabolomics point of view to find metabolic alterations. And, to evaluate if the pattern found at the acute state remains even after 6 months.

## 2. Results

### 2.1. Baseline Characteristics of the Study Population

Thirty patients with acute DVT were included, with a mean age of 44 ± 12.5 years. The prevalence of gender was female (21), and the BMI was on average 29.9 (26.0–31.7) kg/m^2^, higher than the HIs 25.0 (21.3–28.2). Among 30 patients, 6 (20%) had hypertension and 3 (10%) had dyslipidemia. In addition, 6 (20%) patients presented a previous episode of DVT (more than 3 years). Nineteen patients presented with provoked DVT in the presence of major and minor risk factors described in Table 1. And 11 patients did not present any related risk. Thus, 27 patients were receiving anticoagulation treatment at the time of inclusion: 17 (57%) with rivaroxaban, 8 (27%) with fractionated heparin, 1 (3%) with dabigatran, and 1 (3%) with warfarin.

Of the 30 patients included in the acute phase, we were unable to repeat the second collection in 9 patients (30%). The mean interval between the 1st and 2nd collections was 7.0 ± 1.8 months. Twelve patients were still taking anticoagulants, without any episodes of recurrence (Table 2). According to the Villalta PTS classification, 12 (57%) patients did not present this complication, 6 (28%) were mild, 2 (9%) were moderate, and 1 (5%) was severe, Table 2. The Clinical summary of DVT patients is available in Table S1.

### 2.2. Acute DVT Patients Exhibit Elevated Inflammatory Cytokines

It was seen that increased levels of molecules in the context of immunology and cellular signaling, such as IL-6, IL-8, and PDGF-AB/BB in acute DVT (Table 3). Glycoprotein, P-selectin, involved in leukocyte adhesion, was increased in DVT samples. IL-10 was not detected by the commercial kit.

### 2.3. Pro-Inflammatory Cytokines Are Decreased at DVT > 6 Months, but Adhesion Molecules Are Elevated

There was a decrease in cytokines after 6 months of sample collection, significantly for P-selectin. Adhesion molecules such as sVCAM-1 and sICAM-1 are increased in chronic DVT (Table 4).

### 2.4. Metabolomic Data Analysis and Multivariate Statistical Analysis

**Figure 1:**
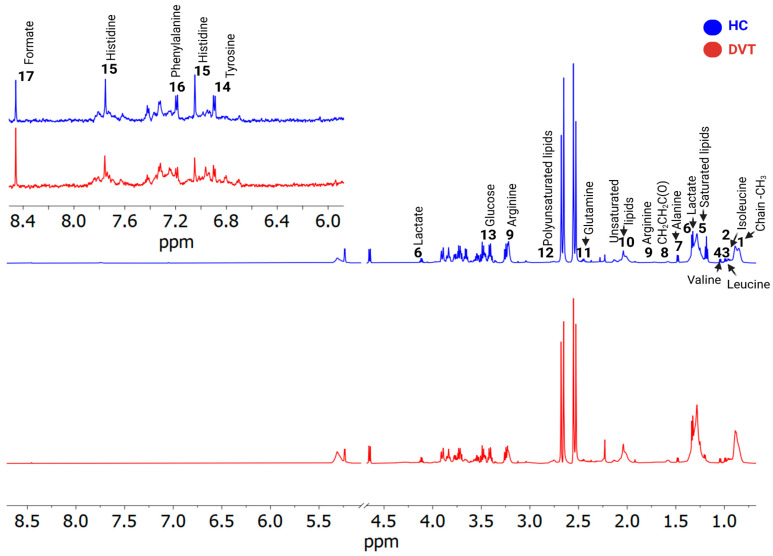
^1^H-NMR (cpmg1d) spectra illustrate differences between two samples—deep venous thrombosis (DVT, shown in red) and a healthy individual (HC, blue). The identified metabolites were: 1. Fatty acyl chain -CH_3_; 2. Isoleucine; 3. Leucine; 4. Valine; 5. Saturated lipids CH_3_(CH_2_)_n_; 6. Lactate; 7. Alanine; 8. Fatty acyl chain CH_2_CH_2_C(O); 9. Arginine; 10. Unsaturated lipids CH_2_CH=; 11. Glutamine; 12. Polyunsaturated lipids (bis-allylic); 13. Glucose; 14. Tyrosine; 15. Histidine; 16. Phenylalanine, and 17. Formate.

**Figure 2:**
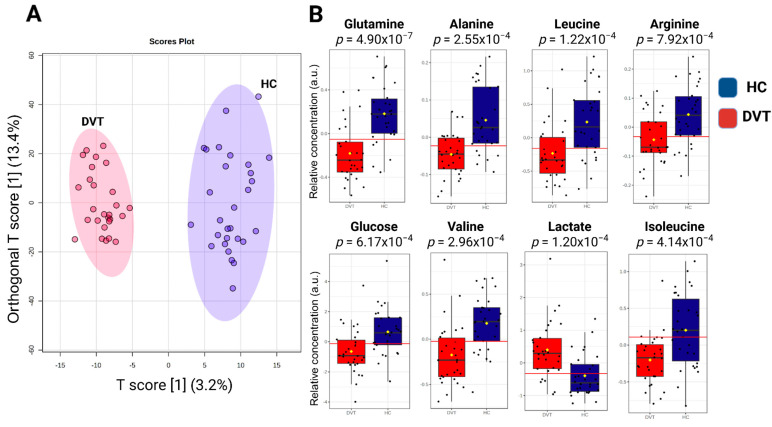
Illustration of the OPLS-DA results: (**A**) Score plot showing the discrimination between acute DVT patients and healthy individuals based on metabolomic profiles. The model is defined by the predictive component T score [1] (3.2%) and the orthogonal component Orthogonal T score [1] (13.4%), which together represent the variance captured in the dataset. The axes indicate the percentage of variance explained by each component. (**B**) Relative metabolite levels (measured as peak intensities) according to the top-eight metabolites with the highest variable importance in projection (VIP) scores. The yellow dot represents the mean value of each group within the boxplot.

A total of 17 known metabolites were identified using ^1^H-NMR T_2_-edited data (Figure 1). For the chemical shifts’ assignments, see Table S2. Based on orthogonal partial least squares discriminatory analysis (OPLS-DA), distinct sample clustering patterns associated with DVT patients and controls can be observed (Figure 2A). Through receiver operating characteristic (ROC) analyses comparing the HIs (healthy control individuals) and DVT, we observed a significant alteration in the levels of glutamine, alanine, valine, lactate, leucine, glucose, isoleucine, and arginine (Figure 2B), with AUC values exceeding 0.74 and *p*-value < 0.05 (Supplementary Figure S1). For qualitative comparison, representative ^1^H-NMR spectra from the control and DVT groups were overlaid without vertical offset, highlighting key metabolites that differed significantly between groups (Supplementary Figure S2).

**Figure 3:**
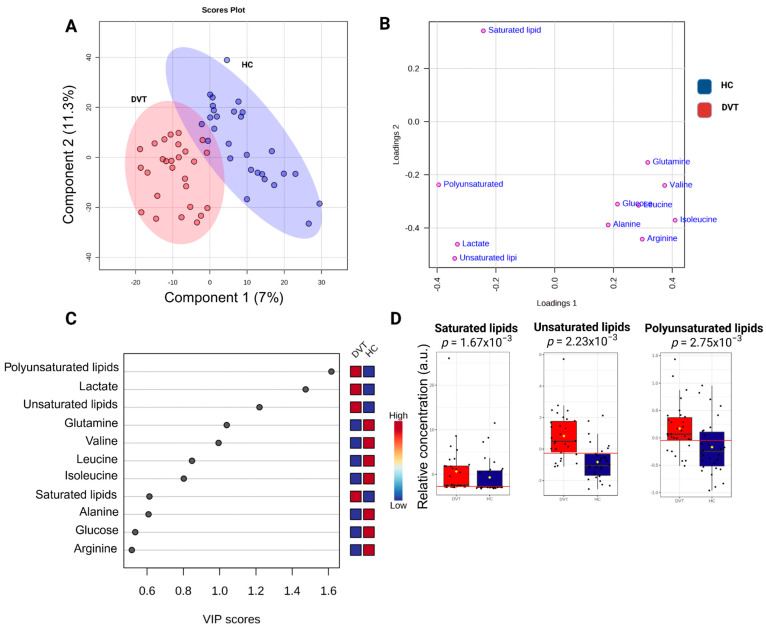
Illustration of the PLS-DA results comparing deep venous thrombosis (DVT) patients and healthy controls: (**A**) Score plot showing sample distribution along Component 1 (7%) and Component 2 (11.3%), highlighting group discrimination based on metabolic profiles. The percentages of variance shown on each axis represent the proportion of the dataset’s total variability captured by each component. (**B**) Loading plot showing the contribution of individual metabolites to model separation. (**C**) Variable importance in projection (VIP) plot indicating the metabolites with the highest discriminatory power. (**D**) Variation in lipid metabolite levels (measured as peak intensities) according to their VIP scores. The yellow dot represents the mean value of each group within the boxplot. Cross-validation results: accuracy = 0.93, R^2^ = 0.91, Q^2^ = 0.70.

A partial least squares-discriminant analysis (PLS-DA) was conducted on all ^1^H-NMR sample data to uncover DVT features compared to healthy individuals, as depicted in Figure 3. Figure 3B is a visual representation of the weights assigned to each metabolite in the PLS-DA model, based on their contribution to the first two latent variables. The variable importance in projection, VIP graph (Figure 3C), was used to show the contribution of the serum variables in the model construction and variation of their levels within the DVT and HI samples. Notably, when considering lipid classes (Figure 3D), we observed the greatest contribution of unsaturated lipids (AUC: 0.833) and polyunsaturated lipids (AUC: 0.721) in comparison to saturated lipids (AUC: 0.69) for distinguishing the DVT samples from HI. In our ^1^H-NMR analysis, we distinguished saturated, unsaturated, and polyunsaturated lipids based on their characteristic chemical shifts. Saturated lipid signals were assigned to the methylene protons at δ 1.24 (–CH_2_–), while unsaturated lipid signals were identified at δ 2.04, corresponding to allylic methylene protons (–CH_2_–CH=CH–) adjacent to double bonds. Additionally, polyunsaturated lipids were identified by the presence of bis-allylic methylene protons (–CH=CH–CH_2_–CH=CH–) at δ 2.74.

**Figure 4:**
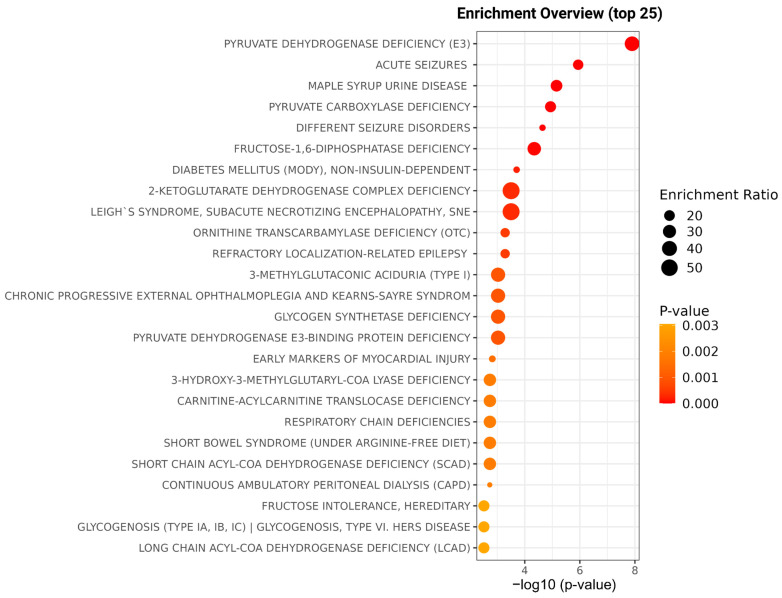
Enrichment overview illustrating the top 25 metabolic pathways significantly altered in acute DVT compared with healthy individuals. The *x*-axis represents the statistical significance of pathway enrichment expressed as –log_10_ (*p*-value), while the *y*-axis lists the identified pathways. The size of the circles corresponds to the enrichment ratio, and the color gradient reflects the *p*-value intensity (from orange to red, indicating increasing significance). The main pathways identified include pyruvate dehydrogenase deficiency, glycogen synthase deficiency, and respiratory chain deficiencies, highlighting disturbances in energy metabolism associated with DVT.

DVT can strongly influence metabolite levels due to inhibited or activated specific metabolic pathways, which reflect and orchestrate the body’s response to unusual events, like the formation of clots. DVT can also be caused by some events in unusual metabolite concentrations. Therefore, enrichment analysis of overall metabolite level alterations was performed to identify metabolic pathways associated with DVT and to provide insights into biological mechanisms potentially linked to thrombus formation or resulting from the thrombotic process (Figure 4). Gluconeogenesis, glycogen synthase deficiency, and respiratory chain deficiencies can be pointed to as the main pathways for the studied DVT.

**Figure 5:**
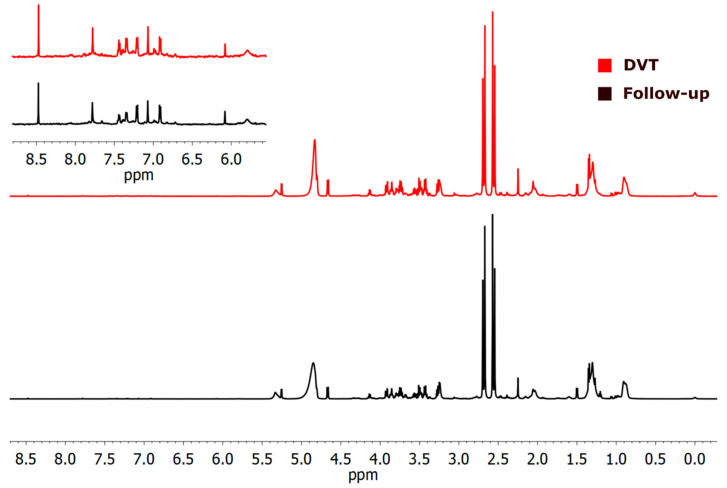
^1^H-NMR (cpmg1d) spectra of the two studied cases illustrate differences between deep venous thrombosis (DVT) and the follow-up group. The DVT sample is shown in red in the upper panel. The aromatic region from 5.9 to 8.6 ppm is 10× amplified, in indent.

**Figure 6:**
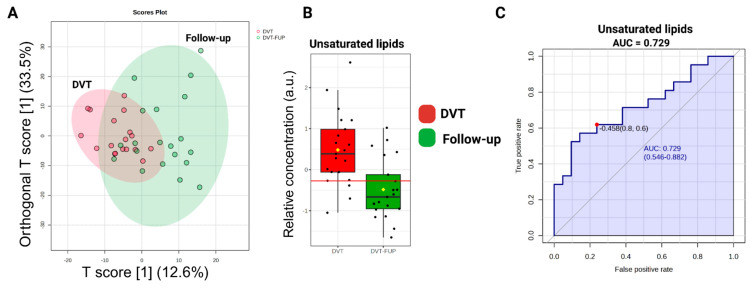
(**A**) Score graph illustrating the OPLS-DA results (Orthogonal T score [1] (33.5%) and T score [1] (12.6%)). (**B**) Relative metabolite levels of unsaturated lipids. The yellow dot represents the mean value of each group within the boxplot. (**C**) Area Under the Curve (AUC) values for the identified biomarkers. The AUC highlights the important discriminatory power of the unsaturated lipids (*p* < 0.05) in DVT.

The ^1^H-NMR spectra of DVT and DVT Follow-up individuals are overlaid in Figure 5. The chemometric analyses conducted are depicted in Figure 6. It is noteworthy that a clear separation of groups was not achieved, as indicated by the OPLS-DA plot (Figure 6A). Only statistically significant unsaturated lipids for distinction (with *p* < 0.05) were observed (Figure 6B), with an AUC equal to 0.729 (Figure 6C). According to these data, Figure S3 shows the metabolic difference between HC and follow-up, demonstrating a persistence of metabolomic dysregulation.

## 3. Discussion

Deep vein thrombosis (DVT) results from disturbances in blood composition, flow, or endothelial cells and accounts for up to 90% of PE. Often asymptomatic, it remains underdiagnosed and contributes significantly to mortality, making it a major public health concern [1,2,18,19]. Accurate detection and understanding of its underlying mechanisms are essential for improving patient outcomes. Biomarkers have become indispensable tools in this context, offering faster and more personalized approaches to diagnosis. The D-dimer assay, reflecting ongoing fibrinolysis, is widely used to rule out DVT due to its strong negative predictive value. Over the past decade, additional biomarkers have been investigated to enhance diagnosis and risk stratification. Studies have shown elevated inflammatory cytokines, including IL-6 and IL-8, and increased platelet-derived growth factor (PDGF) in DVT patients [20,21]. More recently, S100A8 has emerged as a potential novel biomarker, and large-scale analyses of multiple biomarkers have demonstrated high predictive accuracy for DVT [22,23].

Metabolomic profiling provides a complementary approach by revealing systemic metabolic alterations that reflect both disease processes and the body’s response. In this study, we examined metabolic changes in acute DVT patients and assessed whether these changes persist six months after the thrombotic event [9,10]. Using a metabonomic approach, we identified significant differences in serum levels of approximately 16 metabolites between patients and healthy individuals. Nearly half of these metabolites showed an area under the curve (AUC) greater than 0.74, highlighting pronounced alterations in glutamine (Gln), alanine (Ala), valine (Val), leucine (Leu), isoleucine (Ile), arginine (Arg), lactate, and glucose.

Glutamine emerged as a central metabolite, with reduced levels in patients. As the most abundant circulating amino acid, glutamine is essential for DNA, ATP, protein, and lipid synthesis and supports pathways involved in cell proliferation and apoptosis [24,25]. Impaired glutamine metabolism, particularly via inhibition of glutaminase (GLS1), reduces endothelial cell proliferation, compromising vascular repair and potentially contributing to thrombosis [26,27]. Notably, glutamine levels remained low six months after the acute phase, suggesting persistent metabolic dysregulation. Branched-chain amino acids (BCAAs: leucine, isoleucine, valine) were also decreased, consistent with their roles in energy production and metabolic signaling. Dysfunction in BCAAs has been associated with increased arterial thrombosis risk [28]. In contrast, lactate levels were elevated, reflecting enhanced anaerobic metabolism likely driven by hypoxia caused by impaired blood flow. Elevated lactate may therefore serve as an indicator of disease severity and treatment response [29,30,31].

Alterations in glycogen metabolism further suggested a shift toward anaerobic energy production, consistent with hypoxia and disrupted glucose homeostasis. These observations align with prior animal studies showing elevated lactate and purine metabolites in erythrocyte-rich thrombi, indicative of active glycolysis within thrombus components [32,33]. Lipid analysis revealed that unsaturated and polyunsaturated lipids were more relevant than saturated lipids for distinguishing patients from healthy controls. Since lipid-lowering therapies are associated with reduced venous thrombosis risk, these findings support a role for lipid metabolism in thrombus formation [34]. Enrichment analysis also highlighted respiratory chain perturbations, consistent with reports of mitochondrial dysfunction and increased reactive oxygen species in cardiovascular pathology [35].

The observed metabolic alterations likely reflect both predisposing factors and consequences of thrombosis. Serum samples were collected after diagnosis, so the profiles capture both changes induced by the thrombotic event—such as hypoxia-driven anaerobic metabolism and inflammatory stress—and pre-existing metabolic variations, including altered energy and amino acid metabolism. Distinguishing causal from consequential metabolites is clinically relevant: predictive biomarkers may indicate disease risk, whereas markers of metabolic activity can monitor disease progression or recovery. Longitudinal studies are needed to clarify these relationships.

Consistent with previous reports, we observed elevated IL-6, IL-8, and PDGF-AB/BB during the acute phase, reflecting active inflammation [36,37]. These cytokines declined over time, indicating resolution of the acute inflammatory response. Nevertheless, persistent metabolic dysregulation, along with increased vascular adhesion molecules (V-CAM and I-CAM), suggests ongoing low-grade endothelial dysfunction or chronic metabolic shifts [27,38]. This study is the first to combine acute-phase metabolomics with follow-up sampling in DVT patients. Previous studies of the acute phase reported differing glutamine levels, likely due to differences in control groups (healthy individuals versus patients with suspected VTE).

In conclusion, integrating metabolomic profiling with inflammatory and adhesion markers provides a comprehensive view of DVT pathophysiology. Acute DVT induces marked alterations in amino acid, energy, and lipid metabolism, some of which persist beyond the acute phase, reflecting chronic metabolic and endothelial changes. Glutamine, BCAAs, lactate, and lipids emerge as key metabolic indicators, while inflammatory markers offer complementary context. These findings highlight the potential of multi-parameter biomarker panels for improving DVT diagnosis, prognosis, and management. Continued research is necessary to elucidate causal mechanisms and refine biomarker-driven strategies for patient care.

### Limitations and Strengths of the Study

A limitation of the study is the sample size, which was constrained by the strict inclusion and exclusion criteria for patients with acute DVT. The difficulty in selecting patients was particularly evident when excluding those with inflammatory conditions. The decision to focus on a restricted patient group was intended to facilitate more in-depth exploratory analyses while minimizing the epidemiological complexities that could hinder the observation of DVT.

Anticoagulant therapy and the uncertainty regarding its actual impact on cytokine release and the observed metabolic profile could not be entirely excluded, although no significant differences in cytokines were observed in the follow-up group based on continued anticoagulation (Supplementary Table S3).

Also, we focused on the characterization of circulating metabolites but did not assess the expression of related enzymes or regulatory molecules, limiting our ability to pinpoint specific alterations in biosynthesis or utilization pathways. Future studies integrating transcriptomics and proteomic analyses will be essential to elucidate the underlying mechanisms and fully interpret the metabolic alterations observed in DVT. Thus, although our pathway enrichment analysis suggested possible involvement of glycogen synthesis and respiratory chain pathways, it is important to emphasize that key intermediates of these pathways were not directly measured in our study.

Lastly, based on the study design, we cannot determine whether the observed metabolic and endothelial changes are a cause or a consequence of the thrombosis and PE. Further studies investigating the impact of PE on metabolomic profiles after the acute phase, as well as comparisons between provoked and unprovoked DVT, are needed.

As strengths, we believe this study is the first to investigate the acute DVT metabolic profile and track its persistence over a six-month follow-up period, with a well-defined control group of healthy individuals matched by gender and age, which provides a more robust comparison baseline compared to some previous studies that used symptomatic controls.

Also, metabolomics analysis successfully identified a set of eight discriminatory metabolites (e.g., glutamine, lactate, BCAAs, glucose) with high accuracy for distinguishing DVT patients from healthy controls. And, the study incorporated the simultaneous evaluation of established inflammatory markers (IL-6, IL-8) and endothelial markers (sVCAM-1, sICAM-1), which provided essential context confirming the active inflammatory and endothelial response during the acute phase and its persistence into the follow-up.

## 4. Methods

### 4.1. Study Population

This experimental study was carried out between July 2019 to September 2021 in compliance with the Declaration of Helsinki, which was previously approved by the local Ethical Committee. Written informed consents were obtained from all participants of this study.

Patients with DVT in the acute phase were recruited by radiologists and vascular doctors’ collaborators who work at health centers in the metropolitan region of Campinas. Thus, patients who attended UNICAMP were also checked daily through the AGHuse system to select those with an acute diagnosis of thrombosis.

The inclusion criteria for this study were female and male individuals aged between 18 and 70 years old with an acute-phase thrombus in the lower limbs (proximal and/or distal), within 30 days of previous Doppler ultrasound confirmation, and under anticoagulant treatment. In addition, patients were considered to have provoked DVT when thrombosis occurred in the presence of 1 or more transient risk factors (major or minor), as described by the International Society of Thrombosis and Hemostasis in 2016 [39]; and those who did not present any precipitating risk factor were classified as unprovoked DVT. Individuals were excluded under the presence of the following conditions: pregnancy or lactation, cancer in the last 5 years, diabetes mellitus, kidney, liver, or inflammatory disease (acute phase), smoking, and alcohol consumption.

For the prospective evaluation group, patients included in the acute phase were contacted within 6 to 12 months of the date of diagnosis of the source event to carry out a new interview and blood collection.

The control group (HI) was composed of healthy individuals, including female and male individuals aged between 18 and 70 years, without a history of previous DVT. Healthy individuals were matched by gender and age with DVT patients.

### 4.2. Blood Collection and Sample Processing

To avoid possible interfering influences, samples from patients and controls were collected on the same day and immediately processed after venipuncture (within 2 h), under the same conditions.

For each person, we collected 2 tubes of 3.2% sodium citrate, for plasma analysis; 1 dry tube for serum analysis; and 1 tube of EDTA (acid ethylenediamine tetraacetic acid) 10% for blood count.

The samples were processed and stored at the Hemostasis Laboratory of the Hemocentro de Campinas/UNICAMP. Overall, citrate was centrifuged at 3500× *g* for 15 min, and plasma was re-centrifuged to obtain platelet-poor plasma (PPP); with dry tubes, it was centrifuged once at the same rotation. Serum and PPP were aliquoted and stored at −80 °C.

### 4.3. Evaluation of Plasma Markers

Plasma samples from acute DVT and its follow-up and HI were analyzed using Luminex (MILLIPLEX^®^ MAP, Milipore, Burlington, MA, USA) and ELISA (Quantikine^®^) technologies based on fluorescence and absorbance measures, respectively. Both methods were performed following the technical guidelines. The analytes measured through Luminex kits (Bio-plex 200, Bio-Rad, Hercules, CA, USA) were: sVEGFR-2 (code HSCRMAG-32K); sICAM-1 and sVCAM-1 (code HAP1MAG-63K); sCD40L, IFN-γ, TNF-α, IL-1β, IL-6, IL8, IL-10, PDGF-AA/AB (code HCYTA-60K). P-selectin was analyzed by ELISA assay (code DPSE00, R&D Systems).

### 4.4. H-NMR Spectroscopy Analyses

For ^1^H-NMR spectra, 250 µL of each serum sample was mixed with 250 µL of deuterium oxide (D_2_O, 99.9%) containing 0.03% trimethylsilyl propanoic acid (TSP, Sigma–Aldrich, Andover, MA, USA) as a chemical shift reference (δ 0.00), and transferred into 5 mm NMR tubes. Spectra were acquired on a Bruker AVANCE III 600 spectrometer (Bruker BioSpin, Karlsruhe, Germany) operating at 600.17 MHz and equipped with a triple-resonance broadband inverse (TBI) probe at 298 K. One-dimensional ^1^H-NMR experiments were performed using two pulse sequences: NOESY-presaturation (noesygppr1d) for water suppression and Carr–Purcell–Meiboom–Gill (CPMG) (cpmgpr1d) for T_2_ filtering to attenuate macromolecular signals. The acquisition parameters for the NOESY sequence were: spectral width = 6602.0 Hz (≈11 ppm), acquisition time = 2.48 s, relaxation delay (D1) = 4.0 s, presaturation frequency = 4.708 ppm, number of scans (ns) = 128, pulse width = 8.75 µs, and receiver gain = 203.0.

For the CPMG sequence, parameters were: spectral width = 8417.4 Hz (≈14 ppm), acquisition time = 3.89 s, relaxation delay (D1) = 4.0 s, number of scans (ns) = 128, pulse width = 8.75 µs, receiver gain = 203.0, and total repetition time (TR) ≈ 7.9 s. Additionally, two-dimensional total correlation spectroscopy (TOCSY) experiments were acquired using the mlevgpphw5 pulse sequence to confirm metabolite assignments and identify spin systems. The TOCSY acquisition parameters were: spectral width = 8403.4 Hz (in both F1 and F2 dimensions), acquisition time = 0.12 s, relaxation delay (D1) = 3.0 s, number of scans (ns) = 200, pulse width = 9.0 µs, receiver gain = 203.0, and data matrix size = 1024 × 128 (zero-filled to 1024 × 1024). All spectra were processed for phase and baseline correction and referenced to the TSP signal (δ 0.00) using MestReNova software, version 16 (Mestrelab Research, Santiago de Compostela, Spain).

### 4.5. Data Analysis: NMR Data Processing and Statistics

1D ^1^H-NMR spectra were processed to correct their phases and baselines. Chemical shifts were referenced to the TSP signal (δ 0.00), using MestReNova software. The metabolites were assigned based on chemical shifts, coupling constants, 2D NMR spectral features, and in concordance with the Human Metabolome Database (HMDB) and BioMagResBank (BMRB) databases. MetaboAnalyst 5.0 was used to develop ROC curves of individual metabolites.

### 4.6. Statistical Analysis

The Shapiro–Wilk test using the GraphPad Prism version 8.0 program tested numerical variables for normality. The comparison between two groups with a Gaussian distribution was performed using the Student’s *t*-test. The variables were treated as non-parametric for comparison between groups and experiments, and comparisons were made using the Mann–Whitney or Kruskal–Wallis tests (independent variables). The significance level was considered *p* ≤ 0.05. The 1H-NMR spectra were normalized by total area and exported to Excel for further processing in MetaboAnalyst, where sample median normalization and mean centering were applied.

## 5. Conclusions

The persistence of metabolic dysregulation (increased lactate, decreased BCAA, glutamine, and glycogen) supports the idea of chronic metabolic stress in these patients. In addition, metabolic alterations in DVT are accompanied by expected inflammatory and endothelial activation markers, supporting the interpretation that persistent metabolic shifts may reflect ongoing endothelial dysfunction or low-grade inflammation due to tissue hypoxia.

## Figures and Tables

**Table 1:** Clinical data of acute DVT patients and HIs.

	Acute DVT (n = 30)	HIs (n = 30)	*p*
**Age** Mean (±SD)	44 ± 12.5	44 ± 12.5	0.81
**Gender**			
Male/Female	9 (30%)/21 (70%)	9 (30%)/21 (70%)	1.0
**BMI** Median (IQR) kg/m^2^	29.9 (26.0–31.7)	25.0 (21.3–28.2)	0.003
**Comorbidities**			
Hypertension	6 (20%)	1 (3.3%)	0.10
Dyslipidemia	3 (10%)	3 (10%)	1.0
Hypothyroidism	3 (10%)	2 (6.6%)	1.0
Others *	8 (26.6%)	3 (10%)	NA
**Recently SARS-CoV-2 infection**	2 (6.6%)	0	0.49
**A previous episode of DVT** More than 3 years	6 (20%)	NA	-
**Number of DVT events per person** Median (min–max)	2 (1–2)	NA	-
**Family history: DVT, stroke, CVA**	12 (40%)	4 (13.3%)	
2nd degree of kinship	6	3	0.47
**DVT LR/LL**	13:18	NA	-
**The time between symptoms and diagnosis**		NA	
1–15 days	23 (76.6%)		-
15–30 days	7 (23.4%)		
**Spontaneous/provoked DVT**	11:19	NA	-
**Major transient risk factor**	8 (42%)	NA	-
Surgery with anesthesia for over 30 min	6		
Immobilization in a hospital with acute illness (>3 days)	2		
**Minor transient risk factor**	12 (58%)	NA	-
Oral contraceptives and hormone replacement therapy	9		
Trauma or fractures	2		
Immobilization outside the hospital—reduced mobility (>3 days)	1		
**An interval of days between DVT diagnosis and blood collection** Median (IQR)	10 (1.0–30.0)	NA	-
**Anticoagulant use**	27 (90%)	1 (3.3%)	<0.0001

* Others: Arthrosis (3); venous insufficiency (1); a herniated disc (1); stroke (1), and gallstone (2). Abbreviations: deep venous thrombosis (DVT); healthy individuals (HIs); standard deviation (SD); interquartile ratio (IQR); body mass index (BMI); deep venous thrombosis of the left leg (DVT LL); deep venous thrombosis of the right leg (DVT LR); not applicable (NA); Cerebrovascular accident (CVA).

**Table 2:** Follow-up in DVT patients.

	Acute DVT (n = 21)	DVT > 6 Months (n = 21)
**BMI** Mean (SD) kg/m^2^	28.3 ± 4.9	28.1 ± 4.8
**Comorbidities**		
Hypertension	4 (19.0%)	2 (9.5%)
Dyslipidemia	1 (4.7%)	2 (9.5%)
Hypothyroidism	1 (4.7%)	1 (4.7%)
**Anticoagulant use**	20 (95.2%)	12 (57.1%)
**The time between acute DVT diagnosis and the second blood collection** Months, mean (±SD)	NA	7 ± 1.8
**PTS**	NA	
Mild		6 (28.5%)
Moderate		2 (9.5%)
Severe		1 (4.7%)
DVT without PTS		12 (57.1%)
**Recurrence of DVT**	NA	0

Abbreviations: deep venous thrombosis (DVT); standard deviation (SD); body mass index (BMI); not applicable (NA); post-thrombotic syndrome (PTS).

**Table 3:** Biomarker levels in DVT patients and HIs.

	*Acute DVT* n = 23	*HI* n = 19	*p* *
IL-1 (pg/mL)	6.3 (IQR 3.1–16.7)	6.7 (IQR 3.1–10.8)	0.79
IL-6 (pg/mL)	3.5 (IQR 1.5–8.9)	0.3 (IQR 0.05–1.4)	0.0001
IL-8 (pg/mL)	1.3 (IQR 0.9–1.8)	0.9 (IQR 0.5–1.3)	0.04
TNF-α (pg/mL)	8.9 (IQR 2.7–15.3)	5.6 (IQR 2.5–9.0)	0.29
IFN-γ (pg/mL)	0.9 (IQR 0.5–1.5)	1.8 (IQR 0.6–3.7)	0.21
sCD40L (pg/mL)	68.8 (IQR 41.6–136.9)	52.7 (IQR 37.4–85.7)	0.22
sICAM-1 (ng/mL)	575.5 (IQR 512.5–822.2)	789.1 (IQR 621.5–962.8)	0.21
sVCAM-1 (ng/mL)	1270 (IQR 1120–1550)	1340 (IQR 1290–1620)	0.31
P-selectin (ng/mL)	35.99 (IQR 33.7–43.5)	34.8 (IQR 31.0–35.8)	0.05
PDGF-AB/BB (pg/mL)	5479 (IQR 3861–7455)	2714 (IQR 1571–4354)	0.004

* *p*-values were calculated using the Mann–Whitney test, and data are expressed by the median and interquartile interval. Abbreviations: DVT: deep venous thrombosis; HI: healthy individual; IL: interleukin; TNF-α: tumor necrosis factor-alpha; IFN-γ: interferon-gamma; sCD40L: soluble ligand CD40; sICAM-1: soluble intercellular adhesion molecule 1; sVCAM-1: soluble vascular cell adhesion molecule 1; sVEGFR-2: soluble vascular endothelial growth factor receptors 2; and PDGF: platelet-derived growth factor, and IQR: interquartile interval.

**Table 4:** Biomarker levels in DVT follow-up.

	Acute DVT n = 19	DVT > 6 Months n = 19	*p* *
IL-1 (pg/mL)	6.3 (IQR 3.1–16.7)	6.2 (IQR 3.1–10.7)	0.81
IL-6 (pg/mL)	3.9 (IQR 1.7–10.6)	1.4 (IQR 0.5–2.9)	0.09
IL-8 (pg/mL)	1.3 (IQR 0.9–1.8)	1.0 (IQR 0.8–2.1)	0.82
TNF-α (pg/mL)	8.9 (IQR 2.7–15.3)	10.2 (IQR 298–13.9)	0.88
IFN-γ (pg/mL)	0.9 (IQR 0.5–1.4)	0.9 (IQR 0.5–4.2)	0.79
sCD40L (pg/mL)	68.8 (47.2–137.1)	81.8 (IQR 38.8–118.8)	0.33
sICAM-1 (pg/mL)	575,536 (IQR 512,487–822,183)	790,820 (IQR 601,059–966,992)	0.0002
sVCAM-1(pg/mL)	1,270,000 (IQR 1,120,000–1,550,000)	1,620,000 (IQR 1,280,000–1,740,000)	0.01
P-selectin (ng/mL)	35.99 (IQR 33.7–43.5)	33.9 (IQR 28.4–35.6)	0.001
PDGF-AB/BB (pg/mL)	5479 (IQR 4175–7035)	5373.5 (IQR 3468–7441.0)	0.79

* *p* values were calculated using the Wilcoxon matched-pairs test, and data are expressed by the median and interquartile interval. Abbreviations: DVT: deep venous thrombosis; HI: healthy individual; IL: interleukin; TNF-α: tumor necrosis factor-alpha; IFN-γ: interferon-gamma; sCD40L: soluble ligand CD40; sICAM-1: soluble intercellular adhesion molecule 1; sVCAM-1: soluble vascular cell adhesion molecule 1; sVEGFR-2: soluble vascular endothelial growth factor receptors 2; and PDGF: platelet-derived growth factor, and IQR: interquartile interval.

## Data Availability

The datasets generated and/or analyzed during the current study are available in the REDcap repository and are available from the corresponding author on reasonable request.

## Supplementary Materials

The following supporting information can be downloaded at: https://www.mdpi.com/article/10.3390/ijms262311345/s1.

## Abbreviations

^1^H-NMR	Proton Nuclear Magnetic Resonance
Ala	Alanine
Arg	Arginine
ATP	Adenosine Triphosphate
AUC	Area Under the Curve
BCAAs	Branched-Chain Amino Acids
BMI	Body Mass Index
DNA	Deoxyribonucleic Acid
DVT	Deep Venous Thrombosis
EC	Endothelial Cells
EDTA	Ethylenediaminetetraacetic Acid
Gln	Glutamine
HC	Healthy Controls
HMDB	Human Metabolome Database
TOCSY	Total Correlation Spectroscopy experiments
ICAM-1	Intercellular Adhesion Molecule-1
IFN-γ	Interferon-Gamma
Ile	Isoleucine
IL-1β	Interleukin-1 Beta
IL-6	Interleukin-6
IL-8	Interleukin-8
IL-10	Interleukin-10
Leu	Leucine
Luminex	Multiplex Assay Technology
MetaboAnalyst	Metabolomics Data Analysis Tool
mtDNA	Mitochondrial DNA
PDGF-AB/BB	Platelet-Derived Growth Factor AB/BB
PPP	Platelet-Poor Plasma
P-selectin	Platelet Activation Marker
ROC	Receiver Operating Characteristic
ROS	Reactive Oxygen Species
sCD40L	Soluble CD40 Ligand
sICAM-1	Soluble Intercellular Adhesion Molecule-1
sVCAM-1	Soluble Vascular Cell Adhesion Molecule-1
sVEGFR-2	Soluble Vascular Endothelial Growth Factor Receptor-2
TBI	Triple Resonance Broadband Inverse
TSP	Trimethylsilyl Propanoic Acid
Val	Valine
VTE	Venous Thromboembolism
VCAM-1	Vascular Cell Adhesion Molecule-1
