# Supplementary material for: Accessing Altered Metabolic Profile in Acute Deep Vein Thrombosis Through Nuclear Magnetic Resonance Spectroscopy
Source: Int J Mol Sci. 2025 Nov 24;26(23):11345. doi: 10.3390/ijms262311345 (PMC12691825; doi:10.3390/ijms262311345)
Supplement: Supplementary file 1 [file ijms-26-11345-s001.zip › ijms-3961251-supplementary.pdf]

Accessing altered metabolic profiles in Acute Deep Vein Thrombosis through Nuclear Magnetic Resonance Spectroscopy

Letícia Queiroz da Silva[1], Thyerre Santana Da Costa[2], Lucas Gelain Martins[2], Silmara Aparecida de Lima Montalvão[3], Stephany Cares Huber[3], Sandra Martins Silva Soares[3], Ljubica Tasic[2] and Joyce Maria Annichino-Bizzacchi[3**]*

Author affiliations:

1. Hemocentro, Haemostasis and Inflammation Laboratory, Universidade Estadual de Campinas – UNICAMP, 13083-970, Campinas, Sao Paulo, Brazil
2. Biological Chemistry Laboratory, Institute of Chemistry, Universidade Estadual de Campinas – UNICAMP, 13083-970, Campinas, Sao Paulo, Brazil
3. Hemocentro, Haemostasis Laboratory, Universidade Estadual de Campinas – UNICAMP, 13083-970, Campinas, Sao Paulo, Brazil

Corresponding authors:* ljubica@unicamp.br and ** joyce@unicamp.br

KEYWORDS: Deep vein thrombosis, Metabolites, Metabolomics, Profiling, Venous thromboembolism.

Table S1. *Clinical summary of DVT patients*

Case No.	Gender	Age (Years)	BMI kg/m^2	Site	Risk Factors	Blood Collection Follow-Up (Months)	PTS
1	Female	44	29.7	LLL	Surgery (> 30 min)	12	Moderate
2	Female	38	26.3	LLL	Hormonal contraceptive	11	Mild
3	Female	55	32.0	LLL	Hormonal contraceptive	6	Mild
4	Female	23	19.2	LLL	Surgery (> 30 min)	6	Absent
5	Female	32	33.8	LLL	Hormonal contraceptive	6	Absent
6	Female	49	27.2	RLL	Hormonal contraceptive	6	Absent
7	Female	27	31.2	LLL	Hormonal contraceptive	No follow-up	NA
8	Female	28	37.1	LLL	Non-provoked	5	Absent
9	Female	53	32.1	LLL	Surgery (> 30 min)	No follow-up	NA
10	Female	56	31.7	LLL	Non-provoked	No follow-up	Absent
11	Female	32	27.4	LLL	Non-provoked	No follow-up	NA

12	Female	37	21.5	LLL	Non-provoked	7	Absent
13	Male	38	28.7	RLL	Surgery (> 30 min)	8	Mild
14	Male	29	20.5	LLL	Trauma or fractures	12	Absent
15	Male	43	30.7	RLL	Hormonal contraceptive	8	Absent
16	Male	34	34.3	LLL	Non-provoked	No follow-up	NR
17	Female	24	26.8	LLL	Hormonal contraceptive	9	Mild
18	Female	42	24.7	LLL and RLL	Non-provoked	6	Severe
19	Female	41	30.1	RLL	Surgery (> 30 min)	7	Mild
20	Female	64	37.6	RLL	Non-provoked	7	Absent
21	Male	45	26.8	RLL	Non-provoked	7	Absent
22	Male	60	31.1	RLL	Surgery (> 30 min)	6	Mild
23	Male	68	31.0	RLL	Reduced mobility (> 3 days)	No follow-up	NA
24	Male	46	25.7	RLL	Reduced mobility (> 3 days)	7	Absent
25	Female	51	22.9	RLL	Non-provoked	6	Absent
26	Female	54	30.1	LLL	Non-provoked	6	Moderate

**27**	*Male*	*61*	*30.1*	*LLL*	*Non-provoked*	*12*	*NA*
**28**	*Female*	*58*	*20.4*	*RLL*	*Non-provoked*	*11*	*NA*
**29**	*Female*	*58*	*41.0*	*RLL*	*Surgery (> 30 min)*	*6*	*NA*
**30**	*Female*	*40*	*26.1*	*LLL*	*Hormonal contraceptive*	*6*	*NA*

Abbreviations: deep venous thrombosis (DVT); body mass index (BMI); post-thrombotic syndrome (PTS); lower-left limb (LLL); lower-right limb (RLL); not applicable (NA); non-reported (NR).

Table S2. Supplementary table – DVT > 6 months patients still under anticoagulant treatment versus no anticoagulation

	Individuals with anticoagulant N = 9	*Individuals with no anticoagulant N = 10*	*p**
IL-1 (pg/mL)	6.52 (IQR 3.14 – 14.57)	6.26 (IQR 2.32 – 9.22)	0.79
IL-6 (pg/mL)	1.19 (IQR 0.70 – 2.45)	2.80 (IQR 0.33 – 3.68)	0.88
IL-8 (pg/mL)	1.20 (0.80 – 2.17)	0.99 (IQR 0.81 – 1.41)	0.51
TNF-α (pg/mL)	7.53 (2.48 – 16.26)	10.29 (IQR 4.46 – 14.17)	0.71
IFN- γ (pg/mL)	0.85 (IQR 0.46 – 1.2)	1.84 (IQR 0.54 – 24.74)	0.16
sCD40L (pg/mL)	64.95 (IQR 35.18 – 119.1)	91.03 (IQR 38.64 – 127.9)	0.49
sICAM-1(pg/mL)	811288 (IQR 618734 – 930804)	752217 (IQR 582293 – 993496)	0.92
sVCAM-1 (pg/mL)	1645000 (IQR 1210000 – 1825000)	1390000 (IQR 1285000 – 1935000)	0.96
P-selectin (ng/mL)	35.11 (28.42 – 35.58)	32.41 (IQR 28.04 – 37.13)	0.78
PDGF-AB/BB (pg/mL)	4960 (IQR 3426 – 6136)	6029 (IQR 3584 – 8572)	0.31

*p values were calculated using the Mann-Whitney test, and data are expressed by the median and interquartile interval.

Abbreviations: DVT: deep venous thrombosis; HI: healthy individual; IL: interleukin; TNF-α: tumor necrosis factor-alpha; IFN-γ: interferon-gamma; sCD40L: soluble ligand CD40; sICAM-1: soluble intercellular adhesion molecule 1; sVCAM-1: soluble vascular cell adhesion molecule 1; sVEGFR-2: soluble vascular endothelial growth factor receptors 2; and PDGF: platelet-derived growth factor, and IQR: interquartile interval.

Table S3. ^{1}H-NMR chemical shift assignments for the metabolites

s, singlet; d, doublet; t, triplet; q, quartet; m, multiplet; dd, doublet of doublets

**Peak**	**Metabolite**	**Moieties**	δ **^{1}H and Multiplicity**
1	Lipids	CH_3	0.81–0.89 (m)
2	Isoleucine	CH_3 (from CH_2-CH_3 terminal group) and CH_3 (branched, from CH-CH_3)	0.91–0.94 (t), 1.01 (d)
3	Leucine	CH_3 (from isobutyl group, terminal methyls), CH (central carbon in isobutyl group)	0.96 (t), 1.72 (m)
4	Valine	γCH_3, βCH, αCH	0.98 (d), 1.04 (d), 3.61 (d)
5	Saturated lipids	$(CH_2)_n$	1.24–1.30 (m)
6	Lactate	βCH_3, αCH	1.33 (d), 4.11 (q)
7	Alanine	βCH_3, αCH	1.47 (d), 3.77 (q)
8	Lipids	CH_2CH_2CO	1.53–1.65 (m)
9	Arginine	γ-CH_2, αCH	1.87-1.94 (m), 3.25 (t)
10	Unsaturated lipids	C**H**$_2$-CH=	1.96–2.09 (m)
11	Glutamine	γ-CH_2, β-CH_2, α-CH	2.12 (m), 2.45 (m), 3.77 (t)
12	Polyunsaturated lipids	–CH=CH–C**H**$_2$–CH=CH– (bis-allylic)	2.74 (m)
13	Glucose	C_1H, C_2H, C_3H,C_4H, C_5H,CH_2-O6	3.24 (dd), 3.39 (m), 3.46 (m), 3.53 (dd), 3.72 (m), 3.83 (m), 3.89 (dd), 4.65 (d), 5.24 (d)
14	Tyrosine	Aromatic ring proton (Ar-H ortho to -OH) and (Ar-H ortho to side chain)	6.89 (m), 7.19 (m)
15	Histidine	Imidazole ring proton (C2-H) and (C5-H)	7.05, 7.74(s)
16	Phenylalanine	Aromatic ring proton (Ar-H ortho to side chain), (Ar-H meta to side chain) and (Ar-H para to side chain)	7.33 (d), 7.37 (m), 7.42 (m)
17	Formate	CH	8.46 (s)

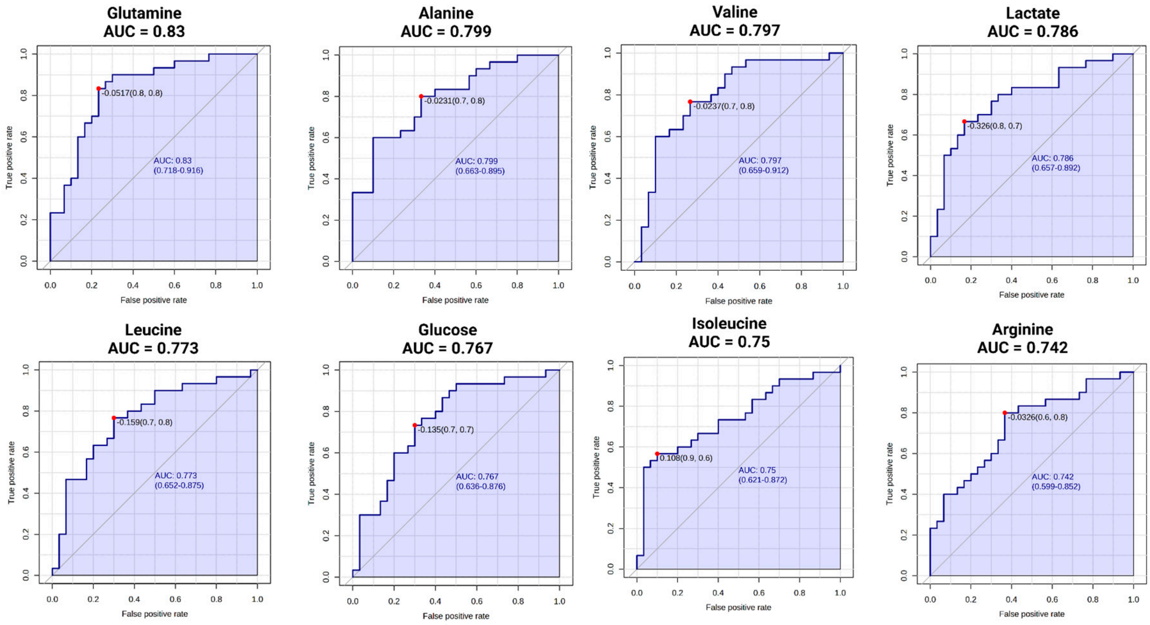

Figure S1. Area Under the Curve (AUC) values for the identified biomarkers.

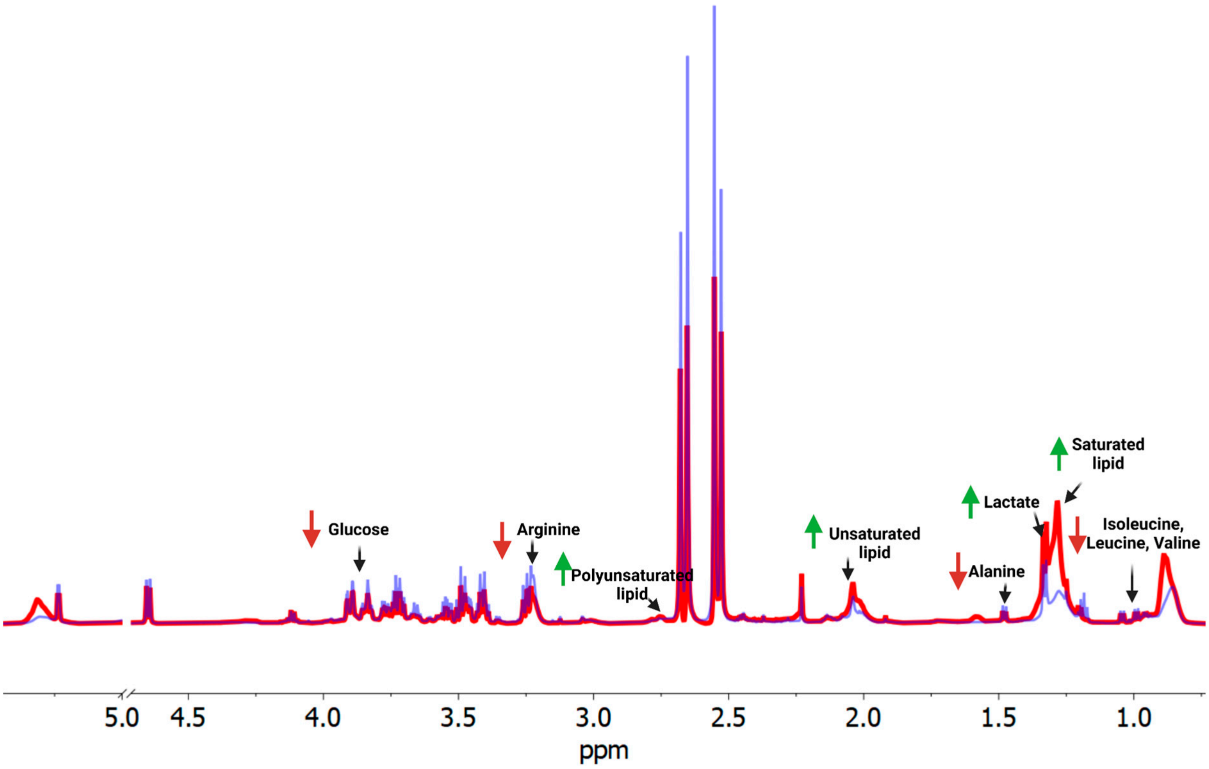

Figure S2. Overlay of representative ^{1}H-NMR spectra (600 MHz, *cpmg1d* sequence) from human serum samples of control (blue) and deep vein thrombosis (DVT, red) groups, displayed without vertical offset. Metabolites showing significant differences between groups are indicated with arrows. Upward arrows (green) represent metabolites that were increased in the DVT group, while downward arrows (red) indicate decreased metabolite levels in the DVT group compared to controls.

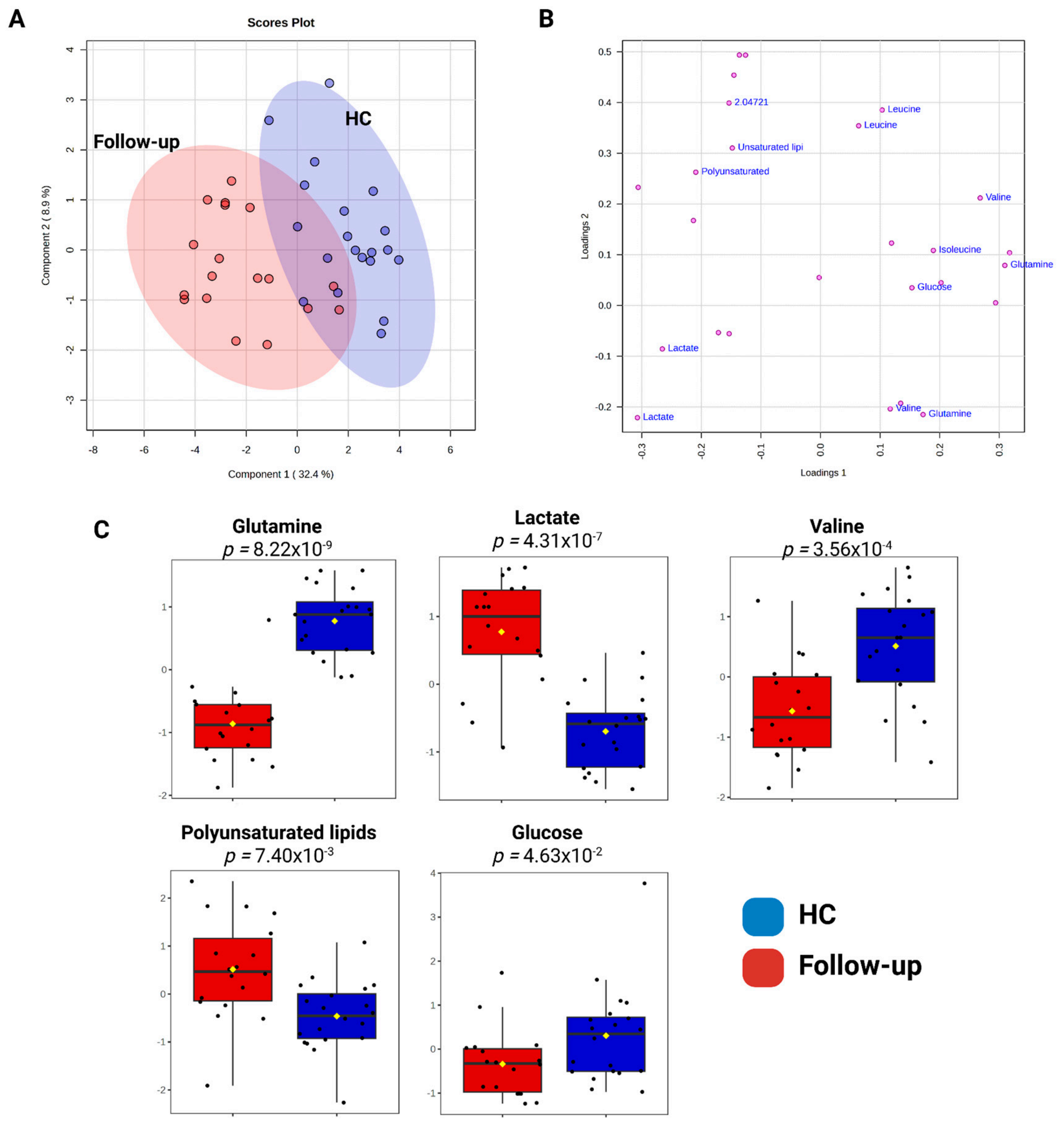

Figure S3. The healthy control (HC) group is shown in blue; the follow-up group in red. **(A)** PLS-DA analysis comparing HC and follow-up groups: Score plot of PC 1 *vs.* PC 2; **(B)** Corresponding loading plot; **(C)** Statistically significant metabolites (p-value < 0.05).
